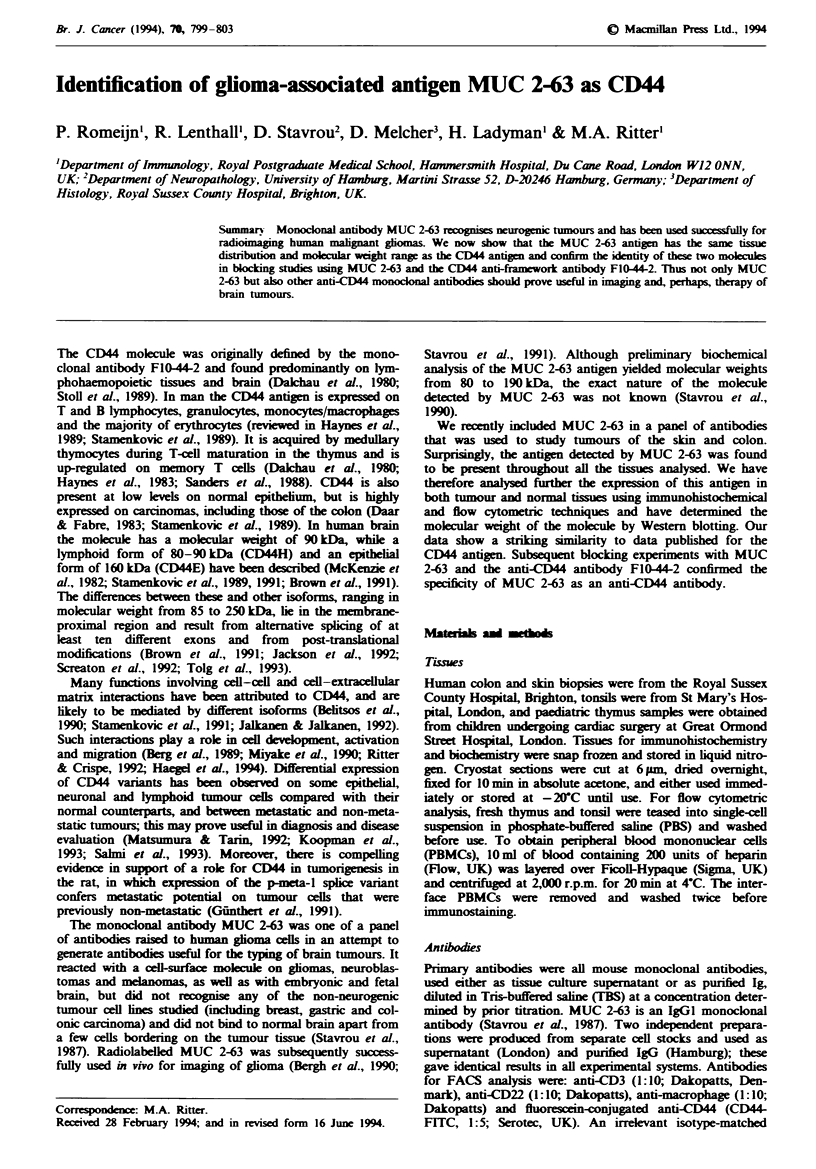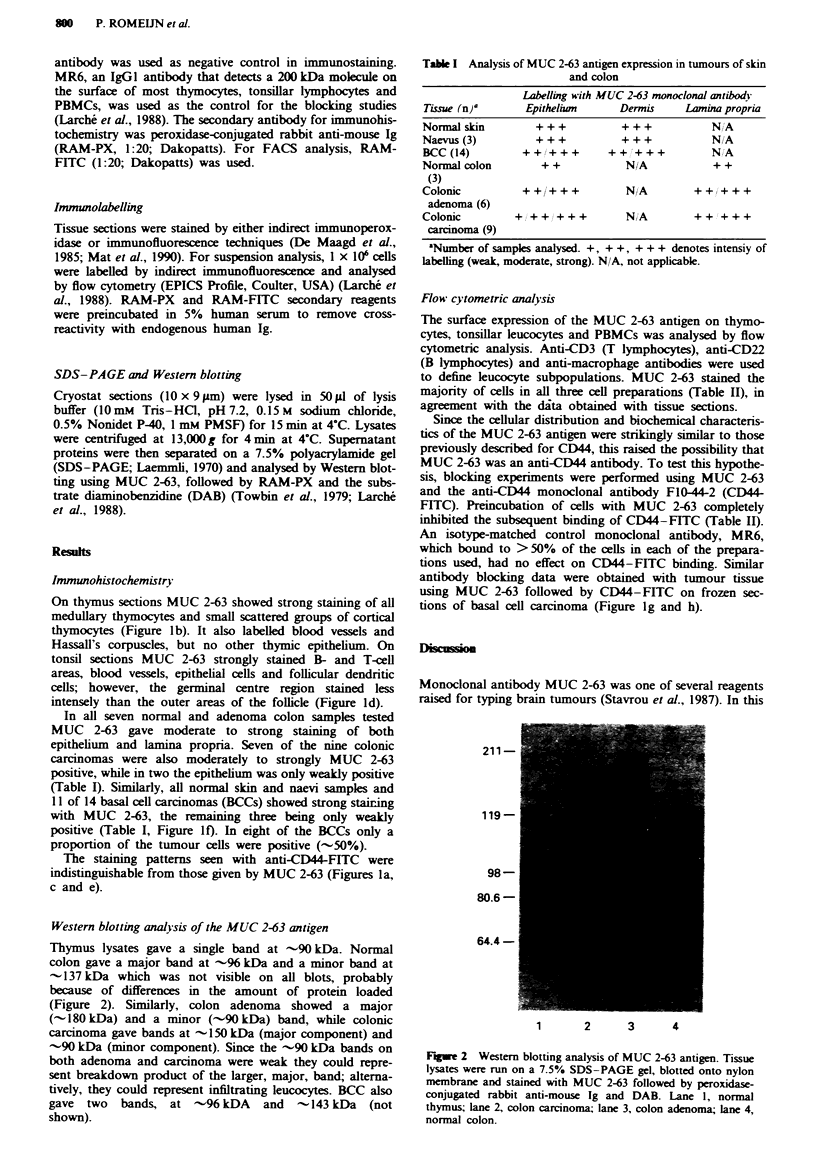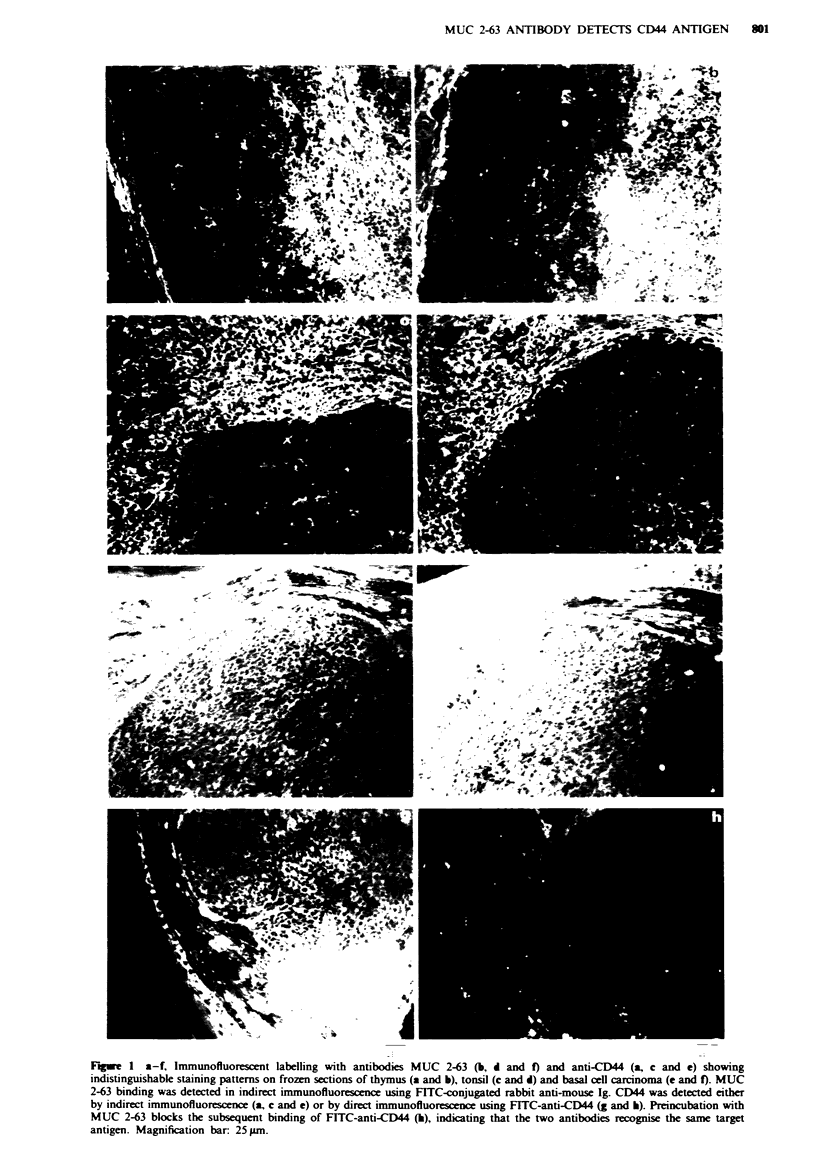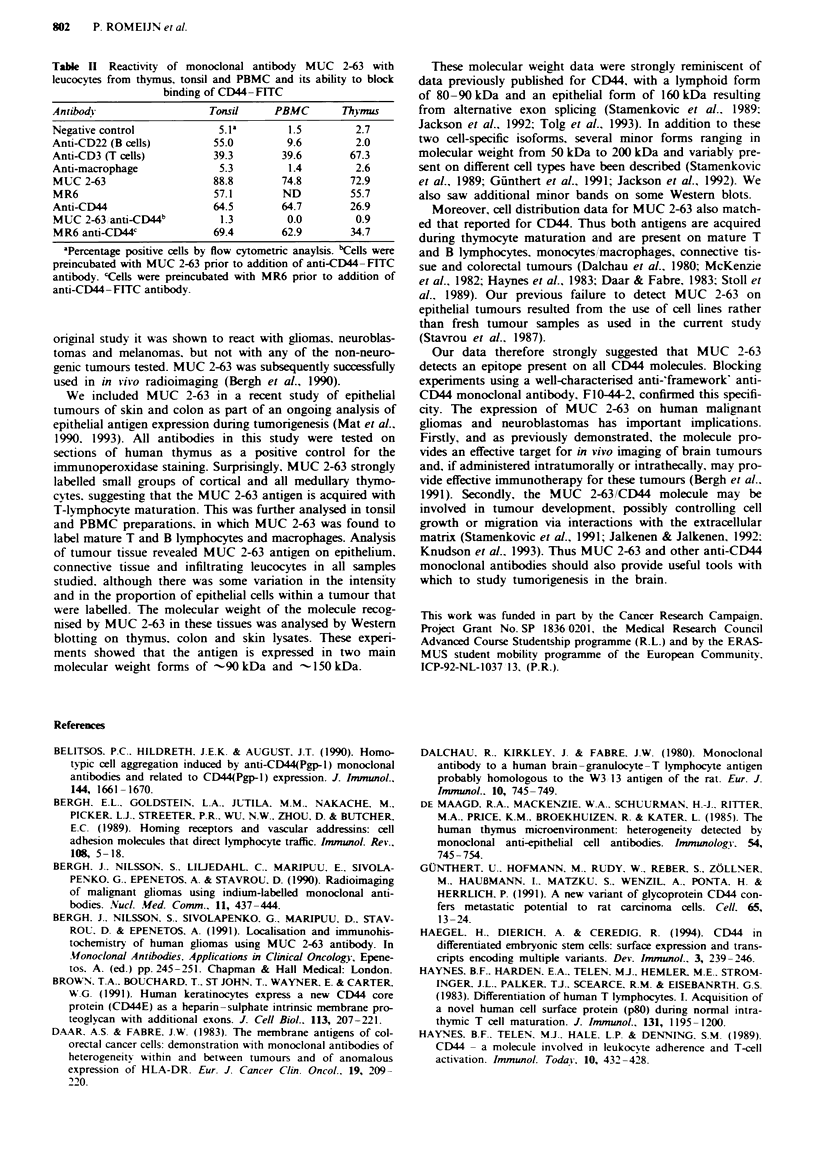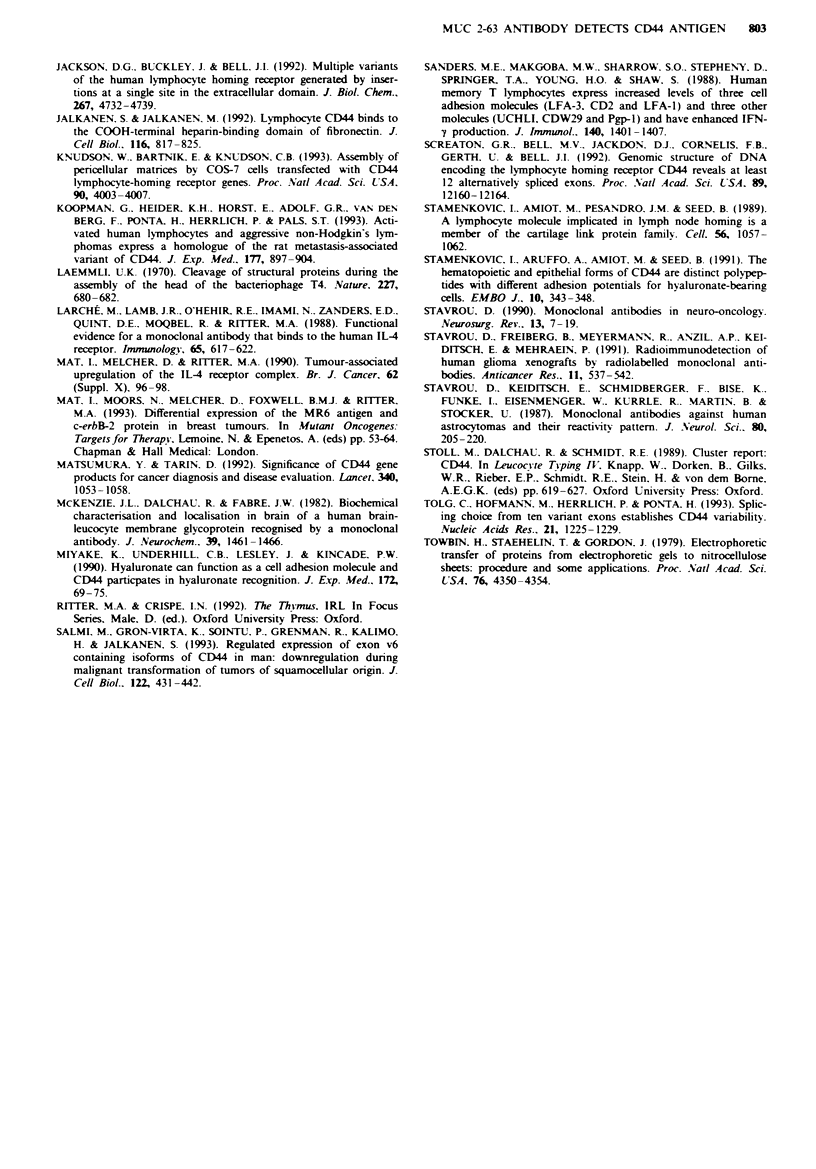# Identification of glioma-associated antigen MUC 2-63 as CD44.

**DOI:** 10.1038/bjc.1994.402

**Published:** 1994-11

**Authors:** P. Romeijn, R. Lenthall, D. Stavrou, D. Melcher, H. Ladyman, M. A. Ritter

**Affiliations:** Department of Immunology, Royal Postgraduate Medical School, Hammersmith Hospital, London, UK.

## Abstract

**Images:**


Br. J. Cancer (1994), 70, 799-803 © Macmillan Press Ltd., 1994

### Identification of glioma-associated antigen MUC 2-63 as CD44

P. Romeijn[1], R. Lenthall[1], D. Stavrou[2], D. Melcher[3], H. Ladyman[1] & M.A. Ritter[1]

[1]*Department of Immunology, Royal Postgraduate Medical School, Hammersmith Hospital, Du Cane Road, London W12 0NN, UK;* [2]*Department of Neuropathology, University of Hamburg, Martini Strasse 52, D-20246 Hamburg, Germany;* [3]*Department of Histology, Royal Sussex County Hospital, Brighton, UK.*

Summary Monoclonal antibody MUC 2-63 recognises neurogenic tumours and has been used successfully for radioimaging human malignant gliomas. We now show that the MUC 2-63 antigen has the same tissue distribution and molecular weight range as the CD44 antigen and confirm the identity of these two molecules in blocking studies using MUC 2-63 and the CD44 anti-framework antibody F10-44-2. Thus not only MUC 2-63 but also other anti-CD44 monoclonal antibodies should prove useful in imaging and, perhaps, therapy of brain tumours.

The CD44 molecule was originally defined by the monoclonal antibody F10-44-2 and found predominantly on lymphohaemopoietic tissues and brain (Dalchau *et al.*, 1980; Stoll *et al.*, 1989). In man the CD44 antigen is expressed on T and B lymphocytes, granulocytes, monocytes/macrophages and the majority of erythrocytes (reviewed in Haynes *et al.*, 1989; Stamenkovic *et al.*, 1989). It is acquired by medullary thymocytes during T-cell maturation in the thymus and is up-regulated on memory T cells (Dalchau *et al.*, 1980; Haynes *et al.*, 1983; Sanders *et al.*, 1988). CD44 is also present at low levels on normal epithelium, but is highly expressed on carcinomas, including those of the colon (Daar & Fabre, 1983; Stamenkovic *et al.*, 1989). In human brain the molecule has a molecular weight of 90 kDa, while a lymphoid form of 80-90 kDa (CD44H) and an epithelial form of 160 kDa (CD44E) have been described (McKenzie *et al.*, 1982; Stamenkovic *et al.*, 1989, 1991; Brown *et al.*, 1991). The differences between these and other isoforms, ranging in molecular weight from 85 to 250 kDa, lie in the membrane-proximal region and result from alternative splicing of at least ten different exons and from post-translational modifications (Brown *et al.*, 1991; Jackson *et al.*, 1992; Screaton *et al.*, 1992; Tolg *et al.*, 1993).

Many functions involving cell-cell and cell-extracellular matrix interactions have been attributed to CD44, and are likely to be mediated by different isoforms (Belitsos *et al.*, 1990; Stamenkovic *et al.*, 1991; Jalkanen & Jalkanen, 1992). Such interactions play a role in cell development, activation and migration (Berg *et al.*, 1989; Miyake *et al.*, 1990; Ritter & Crispe, 1992; Haegel *et al.*, 1994). Differential expression of CD44 variants has been observed on some epithelial, neuronal and lymphoid tumour cells compared with their normal counterparts, and between metastatic and non-metastatic tumours; this may prove useful in diagnosis and disease evaluation (Matsumura & Tarin, 1992; Koopman *et al.*, 1993; Salmi *et al.*, 1993). Moreover, there is compelling evidence in support of a role for CD44 in tumorigenesis in the rat, in which expression of the p-meta-1 splice variant confers metastatic potential on tumour cells that were previously non-metastatic (Günthert *et al.*, 1991).

The monoclonal antibody MUC 2-63 was one of a panel of antibodies raised to human glioma cells in an attempt to generate antibodies useful for the typing of brain tumours. It reacted with a cell-surface molecule on gliomas, neuroblastomas and melanomas, as well as with embryonic and fetal brain, but did not recognise any of the non-neurogenic tumour cell lines studied (including breast, gastric and colonic carcinoma) and did not bind to normal brain apart from a few cells bordering on the tumour tissue (Stavrou *et al.*, 1987). Radiolabelled MUC 2-63 was subsequently successfully used *in vivo* for imaging of glioma (Bergh *et al.*, 1990; Stavrou *et al.*, 1991). Although preliminary biochemical analysis of the MUC 2-63 antigen yielded molecular weights from 80 to 190 kDa, the exact nature of the molecule detected by MUC 2-63 was not known (Stavrou *et al.*, 1990).

We recently included MUC 2-63 in a panel of antibodies that was used to study tumours of the skin and colon. Surprisingly, the antigen detected by MUC 2-63 was found to be present throughout all the tissues analysed. We have therefore analysed further the expression of this antigen in both tumour and normal tissues using immunohistochemical and flow cytometric techniques and have determined the molecular weight of the molecule by Western blotting. Our data show a striking similarity to data published for the CD44 antigen. Subsequent blocking experiments with MUC 2-63 and the anti-CD44 antibody F10-44-2 confirmed the specificity of MUC 2-63 as an anti-CD44 antibody.

### Materials and methods

### *Tissues*

Human colon and skin biopsies were from the Royal Sussex County Hospital, Brighton, tonsils were from St Mary's Hospital, London, and paediatric thymus samples were obtained from children undergoing cardiac surgery at Great Ormond Street Hospital, London. Tissues for immunohistochemistry and biochemistry were snap frozen and stored in liquid nitrogen. Cryostat sections were cut at 6 μm, dried overnight, fixed for 10 min in absolute acetone, and either used immediately or stored at −20°C until use. For flow cytometric analysis, fresh thymus and tonsil were teased into single-cell suspension in phosphate-buffered saline (PBS) and washed before use. To obtain peripheral blood mononuclear cells (PBMCs), 10 ml of blood containing 200 units of heparin (Flow, UK) was layered over Ficoll-Hypaque (Sigma, UK) and centrifuged at 2,000 r.p.m. for 20 min at 4°C. The interface PBMCs were removed and washed twice before immunostaining.

### *Antibodies*

Primary antibodies were all mouse monoclonal antibodies, used either as tissue culture supernatant or as purified Ig, diluted in Tris-buffered saline (TBS) at a concentration determined by prior titration. MUC 2-63 is an IgG1 monoclonal antibody (Stavrou *et al.*, 1987). Two independent preparations were produced from separate cell stocks and used as supernatant (London) and purified IgG (Hamburg); these gave identical results in all experimental systems. Antibodies for FACS analysis were: anti-CD3 (1:10; Dakopatts, Denmark), anti-CD22 (1:10; Dakopatts), anti-macrophage (1:10; Dakopatts) and fluorescein-conjugated anti-CD44 (CD44-FITC, 1:5; Serotec, UK). An irrelevant isotype-matched

Correspondence: M.A. Ritter.

Received 28 February 1994; and in revised form 16 June 1994.

antibody was used as negative control in immunostaining. MR6, an IgG1 antibody that detects a 200 kDa molecule on the surface of most thymocytes, tonsillar lymphocytes and PBMCs, was used as the control for the blocking studies (Larché et al., 1988). The secondary antibody for immunohistochemistry was peroxidase-conjugated rabbit anti-mouse Ig (RAM-PX, 1:20; Dakopatts). For FACS analysis, RAM-FITC (1:20; Dakopatts) was used.

### *Immunolabelling*

Tissue sections were stained by either indirect immunoperoxidase or immunofluorescence techniques (De Maagd et al., 1985; Mat et al., 1990). For suspension analysis, $1 \times 10^6$ cells were labelled by indirect immunofluorescence and analysed by flow cytometry (EPICS Profile, Coulter, USA) (Larché et al., 1988). RAM-PX and RAM-FITC secondary reagents were preincubated in 5% human serum to remove cross-reactivity with endogenous human Ig.

### *SDS-PAGE and Western blotting*

Cryostat sections ($10 \times 9$ µm) were lysed in 50 µl of lysis buffer (10 mM Tris-HCl, pH 7.2, 0.15 M sodium chloride, 0.5% Nonidet P-40, 1 mM PMSF) for 15 min at 4°C. Lysates were centrifuged at 13,000 *g* for 4 min at 4°C. Supernatant proteins were then separated on a 7.5% polyacrylamide gel (SDS-PAGE; Laemmli, 1970) and analysed by Western blotting using MUC 2-63, followed by RAM-PX and the substrate diaminobenzidine (DAB) (Towbin et al., 1979; Larché et al., 1988).

### Results

### *Immunohistochemistry*

On thymus sections MUC 2-63 showed strong staining of all medullary thymocytes and small scattered groups of cortical thymocytes (Figure 1b). It also labelled blood vessels and Hassall's corpuscles, but no other thymic epithelium. On tonsil sections MUC 2-63 strongly stained B- and T-cell areas, blood vessels, epithelial cells and follicular dendritic cells; however, the germinal centre region stained less intensely than the outer areas of the follicle (Figure 1d).

In all seven normal and adenoma colon samples tested MUC 2-63 gave moderate to strong staining of both epithelium and lamina propria. Seven of the nine colonic carcinomas were also moderately to strongly MUC 2-63 positive, while in two the epithelium was only weakly positive (Table I). Similarly, all normal skin and naevi samples and 11 of 14 basal cell carcinomas (BCCs) showed strong staining with MUC 2-63, the remaining three being only weakly positive (Table I, Figure 1f). In eight of the BCCs only a proportion of the tumour cells were positive (~50%).

The staining patterns seen with anti-CD44-FITC were indistinguishable from those given by MUC 2-63 (Figures 1a, c and e).

### *Western blotting analysis of the MUC 2-63 antigen*

Thymus lysates gave a single band at ~90 kDa. Normal colon gave a major band at ~96 kDa and a minor band at ~137 kDa which was not visible on all blots, probably because of differences in the amount of protein loaded (Figure 2). Similarly, colon adenoma showed a major (~180 kDa) and a minor (~90 kDa) band, while colonic carcinoma gave bands at ~150 kDa (major component) and ~90 kDa (minor component). Since the ~90 kDa bands on both adenoma and carcinoma were weak they could represent breakdown product of the larger, major, band; alternatively, they could represent infiltrating leucocytes. BCC also gave two bands, at ~96 kDA and ~143 kDa (not shown).

**Table I** Analysis of MUC 2-63 antigen expression in tumours of skin and colon

| | Labelling with MUC 2-63 monoclonal antibody | | |
|---|---|---|---|
| *Tissue (n)*[a] | *Epithelium* | *Dermis* | *Lamina propria* |
| Normal skin | + + + | + + + | N/A |
| Naevus (3) | + + + | + + + | N/A |
| BCC (14) | + +/+ + + | + +/+ + + | N/A |
| Normal colon (3) | + + | N/A | + + |
| Colonic adenoma (6) | + +/+ + + | N/A | + +/+ + + |
| Colonic carcinoma (9) | +/+ +/+ + + | N/A | + +/+ + + |

[a]Number of samples analysed. +, + +, + + + denotes intensiy of labelling (weak, moderate, strong). N/A, not applicable.

### *Flow cytometric analysis*

The surface expression of the MUC 2-63 antigen on thymocytes, tonsillar leucocytes and PBMCs was analysed by flow cytometric analysis. Anti-CD3 (T lymphocytes), anti-CD22 (B lymphocytes) and anti-macrophage antibodies were used to define leucocyte subpopulations. MUC 2-63 stained the majority of cells in all three cell preparations (Table II), in agreement with the dâta obtained with tissue sections.

Since the cellular distribution and biochemical characteristics of the MUC 2-63 antigen were strikingly similar to those previously described for CD44, this raised the possibility that MUC 2-63 was an anti-CD44 antibody. To test this hypothesis, blocking experiments were performed using MUC 2-63 and the anti-CD44 monoclonal antibody F10-44-2 (CD44-FITC). Preincubation of cells with MUC 2-63 completely inhibited the subsequent binding of CD44-FITC (Table II). An isotype-matched control monoclonal antibody, MR6, which bound to >50% of the cells in each of the preparations used, had no effect on CD44-FITC binding. Similar antibody blocking data were obtained with tumour tissue using MUC 2-63 followed by CD44-FITC on frozen sections of basal cell carcinoma (Figure 1g and h).

### Discussion

Monoclonal antibody MUC 2-63 was one of several reagents raised for typing brain tumours (Stavrou et al., 1987). In this

**Figure 2** Western blotting analysis of MUC 2-63 antigen. Tissue lysates were run on a 7.5% SDS-PAGE gel, blotted onto nylon membrane and stained with MUC 2-63 followed by peroxidase-conjugated rabbit anti-mouse Ig and DAB. Lane 1, normal thymus; lane 2, colon carcinoma; lane 3, colon adenoma; lane 4, normal colon.

Figure 1 a–f, Immunofluorescent labelling with antibodies MUC 2-63 (b, d and f) and anti-CD44 (a, c and e) showing indistinguishable staining patterns on frozen sections of thymus (a and b), tonsil (c and d) and basal cell carcinoma (e and f). MUC 2-63 binding was detected in indirect immunofluorescence using FITC-conjugated rabbit anti-mouse Ig. CD44 was detected either by indirect immunofluorescence (a, c and e) or by direct immunofluorescence using FITC-anti-CD44 (g and h). Preincubation with MUC 2-63 blocks the subsequent binding of FITC-anti-CD44 (h), indicating that the two antibodies recognise the same target antigen. Magnification bar: 25 μm.

**Table II** Reactivity of monoclonal antibody MUC 2-63 with leucocytes from thymus, tonsil and PBMC and its ability to block binding of CD44-FITC

| Antibody | Tonsil | PBMC | Thymus |
|---|---|---|---|
| Negative control | 5.1[a] | 1.5 | 2.7 |
| Anti-CD22 (B cells) | 55.0 | 9.6 | 2.0 |
| Anti-CD3 (T cells) | 39.3 | 39.6 | 67.3 |
| Anti-macrophage | 5.3 | 1.4 | 2.6 |
| MUC 2-63 | 88.8 | 74.8 | 72.9 |
| MR6 | 57.1 | ND | 55.7 |
| Anti-CD44 | 64.5 | 64.7 | 26.9 |
| MUC 2-63/anti-CD44[b] | 1.3 | 0.0 | 0.9 |
| MR6/anti-CD44[c] | 69.4 | 62.9 | 34.7 |

[a]Percentage positive cells by flow cytometric anaylsis. [b]Cells were preincubated with MUC 2-63 prior to addition of anti-CD44-FITC antibody. [c]Cells were preincubated with MR6 prior to addition of anti-CD44-FITC antibody.

original study it was shown to react with gliomas, neuroblastomas and melanomas, but not with any of the non-neurogenic tumours tested. MUC 2-63 was subsequently successfully used in *in vivo* radioimaging (Bergh *et al*., 1990).

We included MUC 2-63 in a recent study of epithelial tumours of skin and colon as part of an ongoing analysis of epithelial antigen expression during tumorigenesis (Mat *et al*., 1990, 1993). All antibodies in this study were tested on sections of human thymus as a positive control for the immunoperoxidase staining. Surprisingly, MUC 2-63 strongly labelled small groups of cortical and all medullary thymocytes, suggesting that the MUC 2-63 antigen is acquired with T-lymphocyte maturation. This was further analysed in tonsil and PBMC preparations, in which MUC 2-63 was found to label mature T and B lymphocytes and macrophages. Analysis of tumour tissue revealed MUC 2-63 antigen on epithelium, connective tissue and infiltrating leucocytes in all samples studied, although there was some variation in the intensity and in the proportion of epithelial cells within a tumour that were labelled. The molecular weight of the molecule recognised by MUC 2-63 in these tissues was analysed by Western blotting on thymus, colon and skin lysates. These experiments showed that the antigen is expressed in two main molecular weight forms of ~90 kDa and ~150 kDa.

These molecular weight data were strongly reminiscent of data previously published for CD44, with a lymphoid form of 80-90 kDa and an epithelial form of 160 kDa resulting from alternative exon splicing (Stamenkovic *et al*., 1989; Jackson *et al*., 1992; Tolg *et al*., 1993). In addition to these two cell-specific isoforms, several minor forms ranging in molecular weight from 50 kDa to 200 kDa and variably present on different cell types have been described (Stamenkovic *et al*., 1989; Günthert *et al*., 1991; Jackson *et al*., 1992). We also saw additional minor bands on some Western blots.

Moreover, cell distribution data for MUC 2-63 also matched that reported for CD44. Thus both antigens are acquired during thymocyte maturation and are present on mature T and B lymphocytes, monocytes/macrophages, connective tissue and colorectal tumours (Dalchau *et al*., 1980; McKenzie *et al*., 1982; Haynes *et al*., 1983; Daar & Fabre, 1983; Stoll *et al*., 1989). Our previous failure to detect MUC 2-63 on epithelial tumours resulted from the use of cell lines rather than fresh tumour samples as used in the current study (Stavrou *et al*., 1987).

Our data therefore strongly suggested that MUC 2-63 detects an epitope present on all CD44 molecules. Blocking experiments using a well-characterised anti-'framework' anti-CD44 monoclonal antibody, F10-44-2, confirmed this specificity. The expression of MUC 2-63 on human malignant gliomas and neuroblastomas has important implications. Firstly, and as previously demonstrated, the molecule provides an effective target for *in vivo* imaging of brain tumours and, if administered intratumorally or intrathecally, may provide effective immunotherapy for these tumours (Bergh *et al*., 1991). Secondly, the MUC 2-63/CD44 molecule may be involved in tumour development, possibly controlling cell growth or migration via interactions with the extracellular matrix (Stamenkovic *et al*., 1991; Jalkenen & Jalkenen, 1992; Knudson *et al*., 1993). Thus MUC 2-63 and other anti-CD44 monoclonal antibodies should also provide useful tools with which to study tumorigenesis in the brain.

This work was funded in part by the Cancer Research Campaign, Project Grant No. SP 1836/0201, the Medical Research Council Advanced Course Studentship programme (R.L.) and by the ERASMUS student mobility programme of the European Community, ICP-92-NL-1037/13, (P.R.).

### References

BELITSOS, P.C., HILDRETH, J.E.K. & AUGUST, J.T. (1990). Homotypic cell aggregation induced by anti-CD44(Pgp-1) monoclonal antibodies and related to CD44(Pgp-1) expression. *J. Immunol.*, **144**, 1661-1670.

BERGH, E.L., GOLDSTEIN, L.A., JUTILA, M.M., NAKACHE, M., PICKER, L.J., STREETER, P.R., WU, N.W., ZHOU, D. & BUTCHER, E.C. (1989). Homing receptors and vascular addressins: cell adhesion molecules that direct lymphocyte traffic. *Immunol. Rev.*, **108**, 5-18.

BERGH, J., NILSSON, S., LILJEDAHL, C., MARIPUU, E., SIVOLAPENKO, G., EPENETOS, A. & STAVROU, D. (1990). Radioimaging of malignant gliomas using indium-labelled monoclonal antibodies. *Nucl. Med. Comm.*, **11**, 437-444.

BERGH, J., NILSSON, S., SIVOLAPENKO, G., MARIPUU, D., STAVROU, D. & EPENETOS, A. (1991). Localisation and immunohistochemistry of human gliomas using MUC 2-63 antibody. In *Monoclonal Antibodies, Applications in Clinical Oncology*, Epenetos, A. (ed.) pp. 245-251. Chapman & Hall Medical: London.

BROWN, T.A., BOUCHARD, T., ST JOHN, T., WAYNER, E. & CARTER, W.G. (1991). Human keratinocytes express a new CD44 core protein (CD44E) as a heparin-sulphate intrinsic membrane proteoglycan with additional exons. *J. Cell Biol.*, **113**, 207-221.

DAAR, A.S. & FABRE, J.W. (1983). The membrane antigens of colorectal cancer cells: demonstration with monoclonal antibodies of heterogeneity within and between tumours and of anomalous expression of HLA-DR. *Eur. J. Cancer Clin. Oncol.*, **19**, 209-220.

DALCHAU, R., KIRKLEY, J. & FABRE, J.W. (1980). Monoclonal antibody to a human brain-granulocyte-T lymphocyte antigen probably homologous to the W3/13 antigen of the rat. *Eur. J. Immunol.*, **10**, 745-749.

DE MAAGD, R.A., MACKENZIE, W.A., SCHUURMAN, H.-J., RITTER, M.A., PRICE, K.M., BROEKHUIZEN, R. & KATER, L. (1985). The human thymus microenvironment: heterogeneity detected by monoclonal anti-epithelial cell antibodies. *Immunology*, **54**, 745-754.

GÜNTHERT, U., HOFMANN, M., RUDY, W., REBER, S., ZÖLLNER, M., HAUßMANN, I., MATZKU, S., WENZIL, A., PONTA, H. & HERRLICH, P. (1991). A new variant of glycoprotein CD44 confers metastatic potential to rat carcinoma cells. *Cell*, **65**, 13-24.

HAEGEL, H., DIERICH, A. & CEREDIG, R. (1994). CD44 in differentiated embryonic stem cells: surface expression and transcripts encoding multiple variants. *Dev. Immunol.*, **3**, 239-246.

HAYNES, B.F., HARDEN, E.A., TELEN, M.J., HEMLER, M.E., STROMINGER, J.L., PALKER, T.J., SCEARCE, R.M. & EISEBANRTH, G.S. (1983). Differentiation of human T lymphocytes. I. Acquisition of a novel human cell surface protein (p80) during normal intrathymic T cell maturation. *J. Immunol.*, **131**, 1195-1200.

HAYNES, B.F., TELEN, M.J., HALE, L.P. & DENNING, S.M. (1989). CD44 - a molecule involved in leukocyte adherence and T-cell activation. *Immunol. Today*, **10**, 432-428.

JACKSON, D.G., BUCKLEY, J. & BELL, J.I. (1992). Multiple variants of the human lymphocyte homing receptor generated by insertions at a single site in the extracellular domain. *J. Biol. Chem.*, **267**, 4732-4739.

JALKANEN, S. & JALKANEN, M. (1992). Lymphocyte CD44 binds to the COOH-terminal heparin-binding domain of fibronectin. *J. Cell Biol.*, **116**, 817-825.

KNUDSON, W., BARTNIK, E. & KNUDSON, C.B. (1993). Assembly of pericellular matrices by COS-7 cells transfected with CD44 lymphocyte-homing receptor genes. *Proc. Natl Acad. Sci. USA*, **90**, 4003-4007.

KOOPMAN, G., HEIDER, K.H., HORST, E., ADOLF, G.R., VAN DEN BERG, F., PONTA, H., HERRLICH, P. & PALS, S.T. (1993). Activated human lymphocytes and aggressive non-Hodgkin's lymphomas express a homologue of the rat metastasis-associated variant of CD44. *J. Exp. Med.*, **177**, 897-904.

LAEMMLI, U.K. (1970). Cleavage of structural proteins during the assembly of the head of the bacteriophage T4. *Nature*, **227**, 680-682.

LARCHÉ, M., LAMB, J.R., O'HEHIR, R.E., IMAMI, N., ZANDERS, E.D., QUINT, D.E., MOQBEL, R. & RITTER, M.A. (1988). Functional evidence for a monoclonal antibody that binds to the human IL-4 receptor. *Immunology*, **65**, 617-622.

MAT, I., MELCHER, D. & RITTER, M.A. (1990). Tumour-associated upregulation of the IL-4 receptor complex. *Br. J. Cancer*, **62** (Suppl. X), 96-98.

MAT, I., MOORS, N., MELCHER, D., FOXWELL, B.M.J. & RITTER, M.A. (1993). Differential expression of the MR6 antigen and c-*erb*B-2 protein in breast tumours. In *Mutant Oncogenes: Targets for Therapy*, Lemoine, N. & Epenetos, A. (eds) pp. 53-64. Chapman & Hall Medical: London.

MATSUMURA, Y. & TARIN, D. (1992). Significance of CD44 gene products for cancer diagnosis and disease evaluation. *Lancet*, **340**, 1053-1058.

MCKENZIE, J.L., DALCHAU, R. & FABRE, J.W. (1982). Biochemical characterisation and localisation in brain of a human brain-leucocyte membrane glycoprotein recognised by a monoclonal antibody. *J. Neurochem.*, **39**, 1461-1466.

MIYAKE, K., UNDERHILL, C.B., LESLEY, J. & KINCADE, P.W. (1990). Hyaluronate can function as a cell adhesion molecule and CD44 particpates in hyaluronate recognition. *J. Exp. Med.*, **172**, 69-75.

RITTER, M.A. & CRISPE, I.N. (1992). *The Thymus*, IRL In Focus Series, Male, D. (ed.). Oxford University Press: Oxford.

SALMI, M., GRON-VIRTA, K., SOINTU, P., GRENMAN, R., KALIMO, H. & JALKANEN, S. (1993). Regulated expression of exon v6 containing isoforms of CD44 in man: downregulation during malignant transformation of tumors of squamocellular origin. *J. Cell Biol.*, **122**, 431-442.

SANDERS, M.E., MAKGOBA, M.W., SHARROW, S.O., STEPHENY, D., SPRINGER, T.A., YOUNG, H.O. & SHAW, S. (1988). Human memory T lymphocytes express increased levels of three cell adhesion molecules (LFA-3, CD2 and LFA-1) and three other molecules (UCHLI, CDW29 and Pgp-1) and have enhanced IFN-$\gamma$ production. *J. Immunol.*, **140**, 1401-1407.

SCREATON, G.R., BELL, M.V., JACKDON, D.J., CORNELIS, F.B., GERTH, U. & BELL, J.I. (1992). Genomic structure of DNA encoding the lymphocyte homing receptor CD44 reveals at least 12 alternatively spliced exons. *Proc. Natl Acad. Sci. USA*, **89**, 12160-12164.

STAMENKOVIC, I., AMIOT, M., PESANDRO, J.M. & SEED, B. (1989). A lymphocyte molecule implicated in lymph node homing is a member of the cartilage link protein family. *Cell*, **56**, 1057-1062.

STAMENKOVIC, I., ARUFFO, A., AMIOT, M. & SEED, B. (1991). The hematopoietic and epithelial forms of CD44 are distinct polypeptides with different adhesion potentials for hyaluronate-bearing cells. *EMBO J.*, **10**, 343-348.

STAVROU, D. (1990). Monoclonal antibodies in neuro-oncology. *Neurosurg. Rev.*, **13**, 7-19.

STAVROU, D., FREIBERG, B., MEYERMANN, R., ANZIL, A.P., KEIDITSCH, E. & MEHRAEIN, P. (1991). Radioimmunodetection of human glioma xenografts by radiolabelled monoclonal antibodies. *Anticancer Res.*, **11**, 537-542.

STAVROU, D., KEIDITSCH, E., SCHMIDBERGER, F., BISE, K., FUNKE, I., EISENMENGER, W., KURRLE, R., MARTIN, B. & STOCKER, U. (1987). Monoclonal antibodies against human astrocytomas and their reactivity pattern. *J. Neurol. Sci.*, **80**, 205-220.

STOLL, M., DALCHAU, R. & SCHMIDT, R.E. (1989). Cluster report: CD44. In *Leucocyte Typing IV*, Knapp, W., Dorken, B., Gilks, W.R., Rieber, E.P., Schmidt, R.E., Stein, H. & von dem Borne, A.E.G.K. (eds) pp. 619-627. Oxford University Press: Oxford.

TOLG, C., HOFMANN, M., HERRLICH, P. & PONTA, H. (1993). Splicing choice from ten variant exons establishes CD44 variability. *Nucleic Acids Res.*, **21**, 1225-1229.

TOWBIN, H., STAEHELIN, T. & GORDON, J. (1979). Electrophoretic transfer of proteins from electrophoretic gels to nitrocellulose sheets: procedure and some applications. *Proc. Natl Acad. Sci. USA*, **76**, 4350-4354.